# Transient lung herniation through a thoracic cage defect: a case report

**DOI:** 10.4076/1757-1626-2-7524

**Published:** 2009-06-12

**Authors:** Kostas Psathakis, Charalampos Mermigkis, Kostas Tsintiris

**Affiliations:** Department of Pneumonology, Army General Hospital of AthensGreece

## Abstract

We report a benign condition of transient lung herniation through a congenital structural defect of the thoracic cage, in a young, otherwise healthy, asymptomatic individual. A brief review of the existing literature on this rare entity is also presented.

## Case presentation

**Figure 1.**
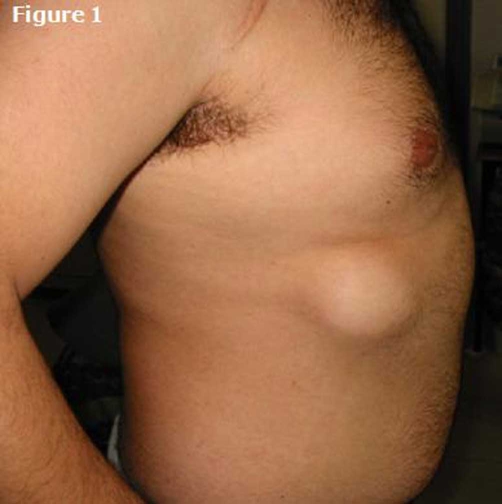
A Valsalva's maneuver causes herniation of the right lung through a “gap” of the thoracic cage.

**Figure 2.**
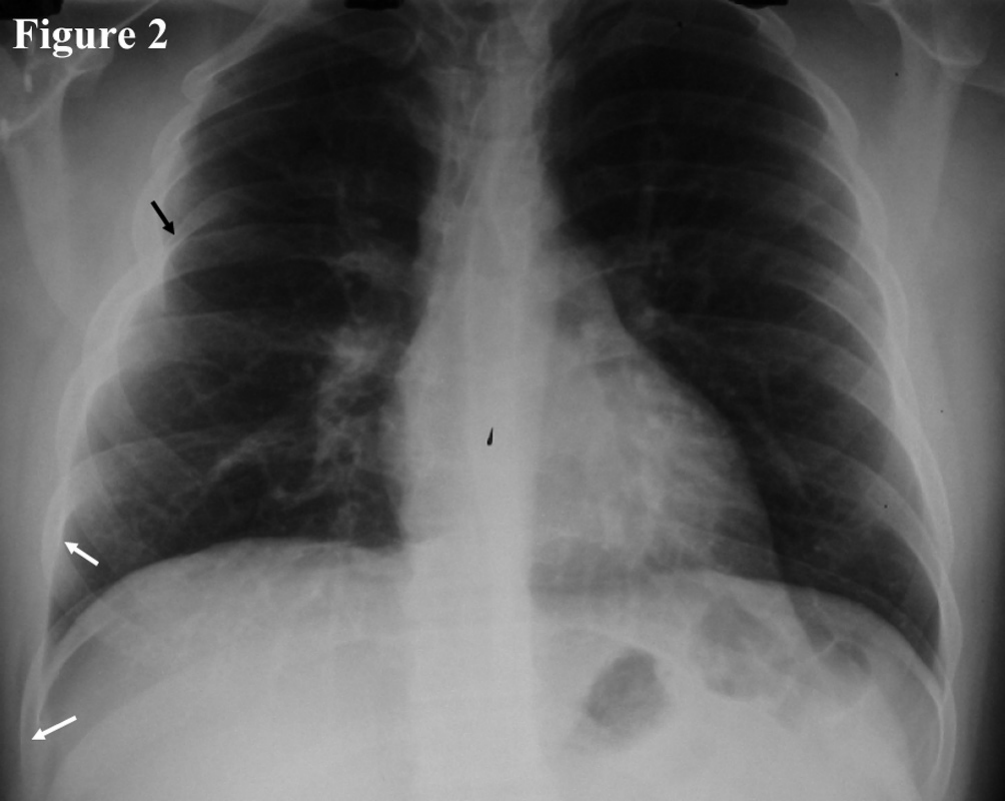
A chest radiography reveals that the anterior portion of the 8^th^ rib comes closer to the 7^th^ rib leaving a gap between the 8^th^ and 9^th^ ribs (white arrows). Note that the 5^th^ right rib has an abnormal configuration as well (black arrow).

A 23-year-old healthy Greek male, came to the outpatient clinic of our hospital for a routine examination for employment. During the examination, he mentioned that his right chest wall protruded whenever he performed a Valsalva's maneuver (Figure 1). He said that it happened since he was a child and he had been informed that it was a benign condition. The rest of the clinical examination, pulse oximetry as well as spirometry were normal. His chest radiography revealed that the 8^th^ and 9^th^ ribs on the right departed from each other leaving a gap (Figure 2). This configuration could explain the transient lung herniation that happened whenever the intrathoracic pressure was increased (e.g. during cough, Valsalva's maneuver etc).

As the patient was asymptomatic, no further intervention was considered.

## Discussion

Herniation of the lung is defined as a protrusion of the lung beyond the normal confines of the thoracic cavity through an abnormal opening of the chest wall [1]. In reference to the underlying mechanism, a sudden growth of intrathoracic pressure is in the background, which can be caused by coughing, emesis, lifting heavy weights; usually combined with attenuation of the thoracic wall [2].

The first case of lung herniation was recorded by Roland in 1499. Since then about 300 patients have been reported, most of them as single case reports. In the majority of cases, the lung herniated through the intercostal space as a result of trauma or after thoracic operation; most of the other hernias were congenital [1,3].

The most widely accepted classification of lung hernia is that of Morel-Lavallee, based on both the anatomic location and etiology. According to their anatomic location pulmonary hernias are classified as diaphragmatic, thoracic (intercostal) and cervical. According to their etiology they are classified as congenital or acquired. Acquired hernias are further classified as spontaneous, traumatic and pathological (secondary to inflammatory or neoplastic processes) [1,4].

A spontaneous lung herniation is more frequent in men and in patients with chronic obstructive pulmonary disease, presumably as a result of chronic coughing and hyperinflation, perhaps combined with long-term steroid administration. The anterior thorax, between the 8^th^ and 9^th^ ribs is the site of predilection for this type of hernias, presumably because of the diminished muscular support at this region [4]. It might be interesting to observe that our patient had a congenital gap of the thoracic cage at this particular intercostal space. The gap combined with the low resistance of the chest wall at this area give a better explanation of the underlying mechanism of the transient lung herniation in this patient.

Postoperative intercostal hernia can develop through disjoining of the intercostal space on the area of thoracotomy or video thoracoscopy [2].

Most lung hernias are asymptomatic. It is likely that a proportion of cases goes unnoticed or gives rise to only minimal symptoms that do not require medical consultation, and therefore the true incidence of the condition is difficult to estimate. This could explain why most pulmonary hernias reported in the literature are not spontaneous but are related to various traumatic conditions [1]. Symptomatic patients usually present with a bulging, crepitant mass protruding through the chest wall either with or without pain. The most important clue is exacerbation of the hernia on Valsalva maneuver, coughing, or straining, with resolution on inspiration or quiet breathing [5].

Incarceration and strangulation, pain, hemoptysis or recurrent infection are the possible complications of the herniation but are rarely observed. In asymptomatic cases conservative management may be sufficient with compressive pads and corsets, and treatment of the underlying condition causing increased intrathoracic pressure. Surgery offers a definitive treatment and is recommended in case of symptoms, interference with everyday activities or for cosmetic reasons [1,4]. It is done by the contraction of the intercostal space with percostal stitches and the reconstruction of the musculature or the implantation of plastic surgical mesh, to cover the gap [2].

In the described patient, the volume of the herniated lung was probably not large enough to affect his every day activities or spirometry. Moreover, since he was a healthy individual with a normal musculature, the lung herniated transiently only when the intrathoracic pressure exceeded the resistant of the muscles that covered the congenital gap of the chest wall. Perhaps for these reasons he had no symptoms since his childhood. Although some authors recommend surgical repair of the defect even in asymptomatic patients [4], we suggest that asymptomatic lung hernias can be left untreated if they do not cause any problems. Obviously, these patients should be followed up and any intervention is justified when symptoms occur [3,5].
